# Antibiotic Prescription During Endodontic Treatment: Knowledge and Practices of Dental Interns in Saudi Arabia

**DOI:** 10.2147/AMEP.S376333

**Published:** 2022-10-17

**Authors:** Mohammed Abdulhai Abuhassna, Hadeel Abdullah Aldajani, Khalil Wassam AlQahtani, Arwa Khader Alzahrani, Deena Abdullah AlAwwad, Oubada Suliman, Mona Talal Rajeh, Sajna Ashraf, Sadeq Ali Al-Maweri

**Affiliations:** 1 Restorative Department, College of Dentistry, Riyadh Elm University, Riyadh, Saudi Arabia; 2 Ministry of Health, Riyadh, Saudi Arabia; 3 Ministry of Health, King Saud Medical City, Riyadh, Saudi Arabia; 4 Faculty of Dentistry, Umm Alqura University, Makkah, Saudi Arabia; 5 College of Dentistry, Riyadh Elm University, Riyadh, Saudi Arabia; 6 Prosthodontics Department, College of Dentistry, Riyadh Elm University, Riyadh, Saudi Arabia; 7 Dental Public Health Department, Faculty of Dentistry, King Abdulaziz University, Jeddah, Saudi Arabia; 8 Department of Oral Medicine and Diagnostic Sciences, Vision Colleges, Riyadh, Saudi Arabia; 9 College of Dental Medicine, QU Health, Qatar University, Doha, Qatar

**Keywords:** antibiotic prescription, knowledge, practices, dental interns

## Abstract

**Background:**

Antibiotics are widely used in dental practice, especially for endodontic infections. The present survey aimed to investigate the knowledge and practices of dental interns in Saudi Arabia regarding antibiotic prescription for endodontic treatment.

**Methods:**

The present online questionnaire-based, cross-sectional study involved dental interns in private and public dental schools, Saudi Arabia. A pre-validated questionnaire was distributed to 900 dental interns via different social media platforms. The questionnaire consisted of 16 close-ended questions related to participants’ demographic data and knowledge and practices of antibiotic prescription in context of endodontic treatments. Data were managed and analyzed using IBM-SPSS version 25, and Chi-square test was used to compare between the groups.

**Results:**

A total of 555 dental interns completed the questionnaire, giving a response rate of 61.1%. Overall, the surveyed participants revealed inadequate knowledge and unnecessary use of antibiotics during endodontic procedures. While majority of the participants (75.3%) correctly identified the first choice of antibiotics during endodontic treatments, a considerable proportion of the participants did not recognize the clinical indications of antibiotics in endodontic patients. Additionally, around one-fifth (18.9%) of the participants were unaware of the potential side effects of the prescribed antibiotics.

**Conclusion:**

The present survey revealed unsatisfactory knowledge and practices of antibiotic prescription in context of endodontic therapy among Saudi dental interns. Therefore, dental schools in Saudi Arabia should address such a gap through updating the curriculum and integrating real-world clinical scenarios using problem-based learning. Additionally, periodic continuous education courses aiming at improving dental professionals’ knowledge about antibiotics and their clinical uses for endodontic therapy are highly encouraged.

## Introduction

Since the discovery of penicillin in 1928 by Fleming, antibiotics have been considered as a significant class of drugs in dental practice.^1^ Unfortunately, however, evidence suggests that a considerable proportion of antibiotic prescriptions in dentistry are unneeded or not optimally recommended, especially in endodontic infections.^1–3^ The misuse and/or overuse of antibiotics by dentists contributes indeed to the worldwide dilemma of antibiotic resistance.^4^ Indications of antibiotics in dental practice may include treating of acute dental infections (such as apical periodontal abscess, cellulitis), in complicated oral and endodontic therapy, and as a prophylaxis in certain systemic diseases.^1^,^2^

In endodontics, debridement of the infected root canal and drainage of the affected soft and hard tissues are the foundations of successful endodontic infection therapy.^5^ The objectives for the treating endodontic infections according to the American Association of Endodontists (AAE) guidelines 2017 are to establish a favorable condition for the lesion to heal through eliminating the pathogenic microorganisms, their by‑products, and pulpal debris from the infected root canal system.^6^ AAE advises antibiotic therapy only in patients suffering from a localized symptomatic apical abscess with systemic manifestations (fever, lymphadenopathy, malaise), progressive infections like cellulitis, soft tissue trauma requiring intervention and extraction, and as a prophylaxis for asymptomatic apical abscess in immunocompromised patients and in predisposing conditions such as endocarditis, otherwise it will be ineffective.^6^ Unfortunately, despite the aforementioned AAE recommendations, the overuse of antibiotics by dentists, especially in treating endodontic infections, is still widespread and likely to increase.^3–5^ Undoubtedly, antibiotic prescribing practice is directly linked to the prescriber’s knowledge and expectations of the patients.^5^ The proper use of antibiotics should be emphasized, and dental professional should be made aware of the indications and consequences of improper antibiotic prescription.^7^ A number of surveys have investigated the knowledge and practices of antibiotic prescription for endodontic infections among dental practitioners worldwide and revealed unsatisfactory knowledge and practices.^8–13^ In Saudi Arabia, data on the awareness and practices of antibiotic prescriptions for endodontic infections among dental professionals are scarce. To date, only two studies documented the awareness and practices of Saudi practicing dentists regarding antibiotics in context of endodontic treatments.^3^,^14^ However, no attempt has been made so far to evaluate the same among Saudi dental students and interns. Such information is critical to identify the gap in the knowledge and design the appropriate educational campaigns to address this issue and prevent the indiscriminate use of antibiotics. Hence, the present study sought to investigate the knowledge and practices of antibiotic prescription during endodontic therapy dental interns in Saudi Arabia.

## Materials and Methods

### Study Design, Participants, and Ethical Approval

The present questionnaire-based, cross-sectional survey was conducted between June and August 2020, following the ethical approval of the Institutional Review Board from the research center at Riyadh Elm University (SRS/2020/32/214/214). All dental interns in public or private dental colleges in Saudi Arabia were eligible to participate. Participation was voluntary, and the participants had the right to withdraw from the study at any time without any penalty. At the beginning of the questionnaire, a cover letter, explaining the aim of the survey and ensuring the confidentiality of data, was included. By clicking on “agree to participate”, the respondent consented to participate in the study, and was then directed to the next pages to complete the questionnaire. Names, emails, or any other personal identifiers were not included in the data collected.

The minimum required sample size was calculated considering 95% confidence level, absolute precision of 5%, and an expected level of knowledge of 50%. The estimated minimum sample size was 380.

### Questionnaire

After reviewing relevant literature and the AAE guidelines on the use of systemic antibiotics in endodontic, a specifically designed free-access Google Forms questionnaire was developed in English language. Prior to distribution of the questionnaire, a pilot study was carried out on 15 dental interns to ensure clarity and reliability. The questionnaire consisted of 16 closed-ended questions, divided into three sections. The first section sought to collect socio-demographic data of the participants including age, gender, nationality, and college. The second section incorporated questions regarding the number of endodontic emergency cases seen per week, different situations to prescribe antibiotics, duration of treatment with antibiotics, first line, second line of treatment and the antibiotic prescribed in penicillin allergy cases, as well as side effects of penicillin/amoxicillin and the course of antibiotic used in cases of spreading infections. Finally, the third section of the survey comprised self-administered questions that aimed to identify the participant’s ability to differentiate the cases in definite need of antibiotic prescription and the cases in need of only local debridement. Hence, this section included three questions involving radiographs and different clinical scenarios opted from actual clinical cases worked on by the main author. The questionnaire link was then sent to all potential participants via various social media platforms, like Twitter and WhatsApp.

### Statistical Analysis

After entries of the data in Microsoft Excel, the IBM-SPSS version 25 (Armonk, NY, USA) was used to analyze the data. Descriptive statistics (such as frequencies and percentages) were calculated, and Chi-square test/Fisher’s exact tests were used to compare between the groups. The significance level was set at P < 0.05.

## Results

Out of the 900 invited interns, only 555 subjects completed the survey, giving a response rate of 61.1%. The demographic characteristics of the participants are presented in Table 1. The majority were females (62.2%) Saudis (92.1%), and from public Universities (51.4%). Around half of the participants (50.6%) encounter less than three endodontic cases, and 36.9% encounter 3–6 cases per week (Table 1).

**Table 1:** Demographic Profile of the Participants (N= 555)

**Gender, n (%)**	
Male	210 (37.8)
Female	345 (62.2)
**Nationality, n (%)**	
Saudi	511 (92.1)
Non-Saudi	44 (7.9)
**University (%)**	
Public	51.34
Private	48.65
**Mean age** (range)	25±2.32 (20–43 years)
**Number of endodontic cases/week**	
<3	281 (50.6)
3–6	205 (36.9)
7–10	37 (6.7)
≥11	32 (5.8)

**Figure 1:**
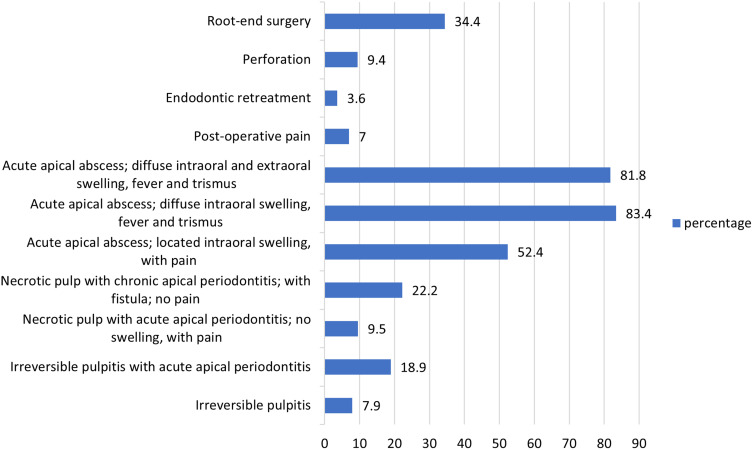
Antibiotic prescribing pattern of participants in specific endodontic cases (%).

The most reported indications of antibiotics in endodontic therapy varied greatly and ranged from as low as 3.6% (endo retreatment) to as high as 83.8% (acute apical) abscess with diffuse intra-oral swelling and systemic manifestations (Figure 1).

Majority of the participants are of the opinion that the proper duration of antibiotic course is 5–7 days, with no significant differences between males and females (P > 0.05). Concerning the first-choice antibiotic prescribed to patients non-allergic to penicillin, amoxicillin 500 mg was the first choice by around two-thirds of the participants, with significant differences between males and females (67.62% vs 80%; p=0.001). Majority of the participants believe that clindamycin is the first choice in patients allergic to penicillin, with no significant differences between the subjects by gender (P > 0.05) (Table 2).

**Table 2:** Participants’ Antibiotic Prescription Patterns by Gender

	Total (%)	Male (%)	Female (%)	p-value
**Duration of antibiotic course**				
3 days	48 (8.6)	18 (8.6)	30 (8.7)	0.650
5–7 days	469 (84.5)	174 (82.9)	295 (85.5)	
10–14 days	24 (4.3)	11 (5.2)	13 (3.8)	
Until symptoms disappear	14 (2.5)	7 (3.3)	7 (2.0)	
**First-choice antibiotic prescribed to patients not allergic to penicillin**				
Amoxicillin 500 mg/3 times	418 (75.3)	142 (67.62)	276 (80)	0.001
Amoxicillin with clavulanic acid 125 mg bid/tid	84 (15.13)	50 (23.81)	34 (9.86)	
No need for antibiotic	53 (9.5)	18 (8.57)	35 (10.14)	
**Second-choice antibiotic prescribed to patients not allergic to penicillin**				
Amoxicillin 500 mg/3 times	129 (23.24)	52 (24.76)	77 (22.32)	0.707
Amoxicillin with clavulanic acid 125 mg bid/tid	359 (64.68)	135 (64.29)	224 (64.93)	
No need for antibiotic	67 (12.07)	23 (10.95)	44 (12.75)	
**First-choice antibiotic prescribed to patients allergic to penicillin**				
Amoxicillin 500 mg/3 times	31 (5.58)	9 (4.29)	22 (6.38)	0.509
Amoxicillin with clavulanic acid 125 mg bid/tid	23 (4.14)	10 (4.76)	13 (3.77)	
Clindamycin 300mg/4 times	501 (90.2)	191 (90.95)	310 (89.86)	
**Using adjunctive antibiotics with adequate debridement and surgical drainage**				
Longest effective course of antibiotics, maximize use of broad-spectrum antibiotics	192 (34.5)	91 (43.3)	101 (29.2)	0.001
Shortest effective course of antibiotic, minimize use of broad-spectrum antibiotics	363 (65.4)	119 (56.6)	244 (70.7)	

**Note**: bid, twice a day; tid, three times a day.

**Figure 2:**
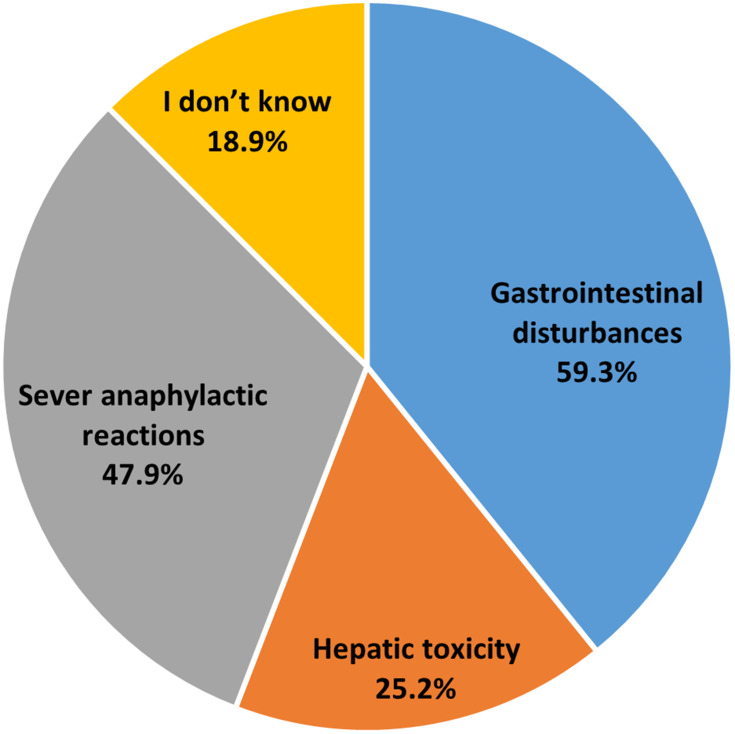
Proportion of antibiotics side effects reported by the participants.

With respect to the question about the potential side effects of penicillin, gastrointestinal disturbances (59%) was the most cited side effect, followed by anaphylactic shock (47.9%), hepatic toxicity (25.2%), while around one-fifth of the subjects were unaware of any potential side effect (Figure 2).

Antibiotic prescribing pattern of participants in different clinical scenarios is presented in Table 3. In case scenario 1 where a medically fit patient exhibited an apical radiolucency in tooth # 12 with history of root canal treatment 8 years ago and no response to Endo-Ice, but tender to percussion and palpation, 81.8% of the participants would not prescribe antibiotics, while only 18.2% felt the need to prescribe antibiotic treatment line. In case scenario 2, where a medically fit patient complaining from puss discharge with sinus tract in relation to tooth # 12 and clinical diagnosis with necrotic pulp, chronic apical abscess, and apical radiolucency, 53.3% would treat the patient with antibiotics, while approximately 46.7% did not want to prescribe antibiotics. In case scenario 3, where a patient with history of prosthetic cardiac valves complaining from tooth # 22 exhibiting an apical radiolucency, clinical diagnosis with pulpal necrosis, and chronic symptomatic apical periodontitis, 65.6% of the participants felt it appropriate to treat such conditions with antibiotics, whereas 34.4% thought it is unnecessary to prescribe antibiotics. The statistical analysis showed no significant difference in the antibiotic prescription pattern between genders on different clinical scenarios (Table 3).

**Table 3:** Antibiotic Prescription on Different Clinical Scenarios

Clinical Scenarios	Gender	Antibiotic Prescription n (%)	No Antibiotic Prescription n (%)	p-value
**Case scenario 1:** Medically fit patient present to your clinic complaining from tooth # 12 exhibits an apical radiolucency. There is history of root canal treatment 8 years ago. There is no response to Endo-Ice, and there is tenderness to percussion and palpation. Is it recommended to prescribe an antibiotic for such case?	Male	43 (20.5)	167 (79.5)	0.278
	Female	58 (16.8)	287 (83.2)	
	Total	101 (18.2)	454 (81.8)	
**Case scenario 2:** Medically fit patient present to your clinic complaining from tooth # 12 exhibits an apical radiolucency and puss discharge with sinus tract. Upon clinical diagnosis the pulpal diagnosis is necrotic, and periapical diagnosis is chronic apical abscess. Is it recommended to prescribe an antibiotic for such case?	Male	115 (54.8)	95 (45.2)	0.599
	Female	181 (52.5)	164 (47.5)	
	Total	296 (53.3)	259 (46.7)	
**Case scenario 3:** Patient present to your clinic with history of prosthetic cardiac valves complaining from tooth # 22 exhibits an apical radiolucency. Upon clinical diagnosis the pulpal diagnosis is necrotic, and periapical diagnosis is chronic symptomatic apical periodontitis. Is it recommended to prescribe an antibiotic for such case?	Male	131 (62.4)	79 (37.6)	0.215
	Female	233 (67.5)	112 (32.5)	
	Total	364 (65.6)	191 (34.4)	

## Discussion

This questionnaire-based, cross-sectional study assessed the knowledge and attitudes of dental interns in Saudi Arabia regarding antibiotic prescription during endodontic treatments. Overall, the surveyed interns showed poor practices and inadequate knowledge on the clinical indications, optimal course duration, and the potential side effects of antibiotics in context of endodontic therapy. It should be noted that the sample size of the present survey (n=555) is threefold greater than that of a 2015 local survey (n=157) by Iqbal,^3^ which is an obvious strength of the present study. Additionally, the present study comprises participants from different private and public Universities in Saudi Arabia compared to the single-institute study by Iqbal et al,^3^ which is another strength. Interestingly, the present survey comprised a high proportion of female participants (62.2%) compared to only 14% in Iqbal et al survey;^3^ this is a very clear reflection of feminization in the profession of dentistry in Saudi Arabia over the recent years.

The onset of endodontic infections is rapid with brief duration and resolves in 3–7 days or less if the cause is treated or removed.^5^ However, there is limited evidence to support the optimal duration of the antibiotic course. The literature implies that shorter course duration for 2–3 days is effective and significantly improves patients’ condition, confirming that a prolonged course of antibiotics may not bestow any additional benefits.^5^,^6^ Disappointingly, only 8.6% of the participants in the present study would prescribe antibiotics for a duration of 3 days, while the majority (84.5%) would prescribe them for a period of 5–7 days. This figure is in line with previous studies conducted among Spanish endodontists,^10^ oral surgeons,^11^ and dental students in Spain.^12^ Additionally, 4.3% and 2.5% of the participants in the present study would prescribe antibiotic therapy for a duration of 10–14 days or until the symptoms subsides, respectively; this indicates the inappropriate use or unawareness of antibiotic abuse. These results confirm inadequate knowledge and lack of up-to-date information regarding the prudent use of antibiotics for endodontic infections among dental interns. Hence, periodic continuous education courses and updating the current curricula should be implemented in order to improve future dentists’ knowledge on the current guideline pertaining antibiotic use in endodontic treatments.

Another key finding of the present study is that the highest percent of antibiotic prescriptions would be in cases of acute apical abscess with diffuse intraoral swelling along with fever and trismus (83.4%), as well as in cases of acute apical abscess with diffuse intra- and extra-oral swelling, fever, and trismus (81%). These figures are similar to findings by a Brazilian survey of 88.1% and 90.1%, respectively.^14^ The antibiotic therapy in the both above mentioned conditions is justifiable and in line with AAE guidelines.^6^

In the present study, 52.4% of the participants would prescribe antibiotics in acute apical abscess with located intraoral swelling and pain; this is highly questionable as necrotic pulp system lacks an effective circulation, and the fundamental treatment for these types of cases is to establish incision and drainage followed by RCT or extraction of the involved tooth to eradicate the cause of infection.^5^,^6^ Additionally, 22.2% of the respondents felt the need of antibiotics in treating necrotic pulp with chronic apical periodontitis with fistula but no pain. The percentage is similar to Rodriguez-Nunez et al study (21.4%),^10^ but contradictory to Segura-Egea et al (60%),^11^ Martin-Jimenez et al (38%)^12^ and Iqbal (46.6%).^3^ It should be noted that such cases can be cured with nonsurgical root canal treatment, and the need for antibiotic is unjustifiable unless there is an acute flare up along with systemic involvement, and an unhealed sinus tract.^4–6^ These findings again confirm the lack of up-to-date and scientific basis for the use of antibiotics in the treatment of endodontic infections.

In the present study 75.3% of the participants chose amoxicillin 500 mg, 3 times/day as the first line of therapeutic antibiotics, which is much higher than that reported by a previous local study (18.3% and 33.7%)^3^ and other international studies (34–47%).^8^,^9^ However, this figure is much lower than that reported by Brazilian endodontists (90.2%)^14^ and Spanish dental students (100%).^12^ According to a recent antibiotic update in endodontics, amoxicillin, and penicillin VK are recommended as the first line of therapeutic antibiotics that dentists must prescribe to non-allergic penicillin patients.^6^,^16^ Being bactericidal, Penicillin VK has great effectiveness, low toxicity, as well as low cost.^15^ The difference between amoxicillin and penicillin VK is that penicillin has relatively narrow spectrum, while amoxicillin has a broader spectrum of antibiotic activity.^15^,^16^ Of note, the development of β -lactamase producing bacteria can result in a significant decrease in the antimicrobial activity of amoxicillin against endodontic pathogens;^16^,^17^ as such, a combination of amoxicillin with clavulanic acid (a β -lactamase inhibitor) is highly recommended.^6^,^15^,^16^ In the present study, 64.7% of our participants prefer this drug combination as the second line of treatment to treat endodontic infection. This figure is much lower than that reported by Turkish dentists (90.3%),^8^ but higher than that reported by Iqbal et al (45.2%),^3^ Rodriguez-Nunez et al (42%),^10^ Martin-Jimenez et al (53%),^12^ and Bolfoni et al (26%).^14^ Additionally, 90.3% of our respondents chose Clindamycin as the first antibiotics in patients with penicillin allergy. These figures were less than that reported by Martin-Jimenez et al (99%),^12^ yet higher than that reported in most of previous studies which ranged from 4.4% to 65%.^3^,^10^,^11^,^13^ Recently, azithromycin was recommended as an effective alternative in treating endodontic infections in b-lactam allergic patients as clindamycin can be related with lethal *Clostridioides difficile* infections.^18^

Interestingly, when the participants were introduced to a real clinical scenario, the majority (81.8%) were against prescribing any antibiotics in case scenario 1, which is very well in line with AAE recommendations.^6^ However, in case scenario 2, 53.3% of the participants would treat their patient with antibiotic in addition to endodontic therapy. This in fact is contradictory to the AAE recommendations, which suggest that adequate debridement of the infected root canals and drainage for both soft and hard tissue are sufficient, and the use of antibiotic is unjustified.^6^ Antibiotic coverage is critical prior to any invasive endodontic therapy in high-risk patients for endocarditis (prosthetic heart valves), as per the AAE recommendations and prophylactic antibiotic guidelines for cardiac valves.^19^ Unfortunately, only 65.6% of the participants recognized this clinical indication as reflected by their intention to prescribe antibiotics for patients with prosthetic cardiac valves complaining from tooth #22 (case scenario 3), indicating an alarming lack of knowledge. Hence, an improvement of dentists’ knowledge on prophylactic antibiotics is highly recommended.

## Study Limitations

The main limitation of the present survey is related to the nature of the sampling (being convenience sampling). This might have led to a selection bias and thus the generalizability of the results is questionable. Additionally, similar to any questionnaire-based survey, the results are based on self-reported responses, and so the responses might not have precisely reflected the real knowledge and practices of the participants.

## Conclusion

In summary, the results reveal that majority of the participants use antibiotic injudiciously during endodontic therapy, reflecting their lack of knowledge on the sensible use and scientific basis for prescription of antibiotics. It is crucial that dental schools in Saudi Arabia make an action to improve dental students’ awareness about antibiotics and their clinical uses in endodontic procedures. This can be achieved through updating the current curriculum and by integrating real-world clinical scenarios through problem-based learning.

## Notes

Open Access funding provided by the Qatar National Library.
